# Rheological Property Criteria for Buildable 3D Printing Concrete

**DOI:** 10.3390/ma12040657

**Published:** 2019-02-21

**Authors:** Hoseong Jeong, Sun-Jin Han, Seung-Ho Choi, Yoon Jung Lee, Seong Tae Yi, Kang Su Kim

**Affiliations:** 1 Department of Architectural Engineering, University of Seoul, 163, Seoulsiripdae-ro, Dongdaemun-gu, Seoul 02504, Korea; besc3217@gmail.com (H.J.); sjhan1219@gmail.com (S.-J.H.); ssarmilmil@uos.ac.kr (S.-H.C.); jjdldbswjd@naver.com (Y.J.L.); 2 Department of Civil Engineering, Inha College, 100, Inha-ro, Nam-gu, Incheon 22212, Korea; yist@inhatc.ac.kr

**Keywords:** 3D printing, concrete, buildability, rheology, mixture design

## Abstract

Fresh concrete used in 3D printing should ensure adequate yield stress, otherwise the printed concrete layer may suffer intolerable deformation or collapse during the printing process. In response to this issue, an analytical study was carried out to derive the initial yield stress and hardening coefficient of fresh concrete suitable for 3D printing. The maximum shear stress distribution of fresh concrete was calculated using a stress transformation equation derived from the equilibrium condition of forces. In addition, the elapsed time experienced by fresh concrete during the printing processes was estimated and was then substituted into the elapsed time-yield stress function to calculate the yield stress distribution. Based on these results, an algorithm capable of deriving both the initial yield stress and the hardening coefficient required for printing fresh concrete up to the target height was proposed and computational fluid dynamics (CFD) analyses were performed to verify the accuracy of the proposed model.

## 1. Introduction

Reinforced concrete (RC) structures have been widely utilized because they are extremely easy and economical to build. However, the disadvantages of the construction method include the facts that it causes an inefficient consumption of materials, problems with the labour force and issues with the construction period in the process of manufacturing the formwork, processing/placing the reinforcement and pouring the concrete [1]. Furthermore, there has been a growing demand for freeform structures that require complicated and heavy formwork [2]. 3D concrete printing technology can be an effective alternative, which can remove the formwork and reduce cost, labour force and the construction period [3]. Therefore, numerous studies have been conducted on the concrete 3D printing technology [4,5,6,7,8,9,10,11,12].

Khoshnevis [4] developed a contour crafting (CC) that uses computer control to exploit the excellent surface-forming capability of trowelling and it is known as the first example of 3D concrete printing. Dini developed a D-shape process that produces a concrete element by spraying a binder onto a layer of powder in a manner similar to powder-bed printing or binder jetting [5]. This printing method is advantageous in that it can produce complicated elements more easily than fused deposition modelling (FDM). Le et al. [6,7] established the key concept of fresh concrete properties, such as buildability, extrudability, open time and the workability of fresh concrete, suitable for concrete 3D printing. In addition, they closely examined the hardened properties, such as the void and dry shrinkage, as well as the mechanical properties of printing concrete. Gosselin et al. [8] used the 3D printing technology to print out a complicated 3D form of concrete members without the use of formworks. Using this particular method, they also developed concrete members with improved performance in heat insulation and sound insulation. Hambach and Volkmer [9] implemented a 3D printing technology able to artificially control fibre orientation in fibre-reinforced Portland cement paste (hereafter referred to as FRC). Their research results confirmed that the flexural strength of the 3D printed FRC varied depending on the printing path and fibre type and it exhibited the highest flexural strength when the volume fraction of carbon fibre was 1% and the 3D printing process progressed along the optimized printing path [9]. Bos et al. [10] summarized the history of the study on additive manufacturing of concrete and addressed the characteristics of 3D concrete printing (3DCP) product geometry and structure. Buswell et al. [11] outlined the technical issues on 3DCP and presented a guide and vision for future research. They also described the effects of the fresh and hardened paste, mortar and concrete properties on geometry of the created object in detail. Labonnote et al. [12] performed mapping study by classifying additive construction into four main categories; material science, engineering, architecture and market analysis.

The constitutive equations of fresh concrete are commonly represented by the Bingham model or the Herschel-Bulkley model [13]. In the 3D printing process, fresh concrete may suffer deformation and collapse when the shear stress generated in concrete exceeds the yield stress [14]. Therefore, in previous studies [15,16,17,18], the maximum shear stress of fresh concrete was calculated based on the equilibrium of forces and compared with the yield stress in order to predict the slump of fresh concrete. Unlike in the slump test, in the 3D concrete printing process, the increase in the yield stress of the fresh concrete should be taken into consideration. The increase in the yield stress according to the elapsed time of fresh concrete is represented by a linear or exponential function [19,20,21]. Perrot et al. [22] predicted the failure of fresh concrete during the printing process by reflecting the increase of the yield stress of fresh concrete over time and they proposed a model for calculating the optimal build-up velocity for concrete printing. They also verified the proposed model through a failure test on fresh concrete. However, in a test conducted by Perrot et al. [22], the fresh concrete showed many deformations prior to its failure and the test results showed that, since these deformations can cause the outputs of other modified forms different from those of the input 3D modelling data, the mixture design of printing concrete should be done to avoid deformation and collapse, rather than failure. Meanwhile, Wangler et al. [23] suggested the printing speed required to prevent collapse or deformation of fresh concrete during layering process. Suiker [24] suggested a mechanical model that could consider buckling collapse and Roussel [25] also proposed a model that reflects the stability failure. Wolfs et al. [26] conducted research on early age mechanical behaviour of 3D printed concrete through numerical simulations and experiments. From their research findings, it was confirmed that the numerical simulations well predict the early age behaviour of 3D printed concrete. Although many researches have been conducted to investigate the behaviour of fresh concrete and to prevent the collapse of layered concrete, it is still necessary to develop analytical models that can evaluate the behaviour of 3D printed concrete in a simple or efficient way without complex numerical simulations.

The purpose of this study is to quickly obtain the initial yield stress and hardening coefficient required to prevent the deformation and collapse of fresh concrete during printing. To this end, the maximum shear stress distribution generated in fresh concrete during the built-up process was calculated using the force equilibrium condition based on the slump prediction model of Murata [15] and the concrete yield stress distribution considering the overtime was calculated using the elapsed time-yield stress equation. In the proposed model, it was assumed that the printed concrete collapses when the maximum shear stress exceeds the yield stress. In other words, the collapse of printed concrete was assumed to be dominated by material failure, excluding stability failure. In addition, ANSYS CFX (ANSYS Inc., Canonsburg, PA, USA), a commercial software program for numerical analysis, was used to verify the reasonability of the proposed model by checking the deformation of layered printed concrete.

## 2. Assumptions

**Figure 1:**
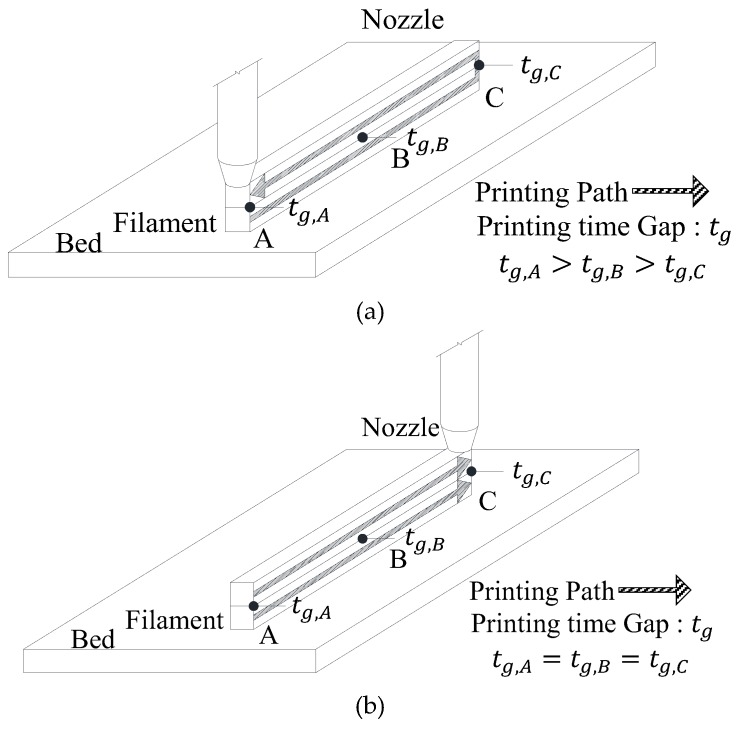
Classification of printing start point at each layer. (**a**) Optimized printing start points for fastest printing speed; (**b**) Selected printing start points closest to specific location.

Figure 1 shows that even in printing work carried out for the same plan, different printing paths are possible depending on the printing start point of each layer (hereafter referred to as the printing start point) can have and the printing start point has a significant effect on the printing time gap. According to the test results conducted by Le et al. [7], as the printing time gap increases, the interfacial bond performance of the hardened 3D printed concrete decreases. This phenomenon was also reported by Feng et al. [27] and Nerella et al. [28]. Therefore, the printing start point needs to be carefully considered before the printing process begins, since it affects the mechanical performance of the hardened 3D printed concrete. This printing start point can be set in the slicing software and is classified into optimized printing start points for the fastest printing velocity (hereafter referred to as OP), in which the closest point after the output of one layer is designated as the printing start point of the next layer in order to minimize the printing time as shown in Figure 1a and the selected printing start points closest to specific location (SP), where the output of one layer, which is returned to the original point after its output, progresses again with the printing start point fixed to a specific location, as shown in Figure 1b. As Figure 1a shows, for the OP, the filaments of each layer exhibit different printing time gaps in the length direction of the printing path. On the other hand, in terms of the SP, the filaments of each layer show the same printing time gap at any point in the length direction of the printing path, as shown in Figure 1b. In this regard, this study assumed the case of SP, which can have the same printing time gap and ensure uniform interfacial bond performance and calculated the yield stress distribution and elapsed time in the printing of the concrete.

**Figure 2:**
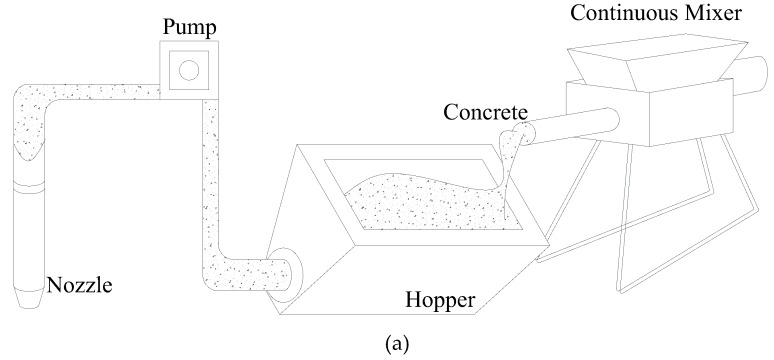

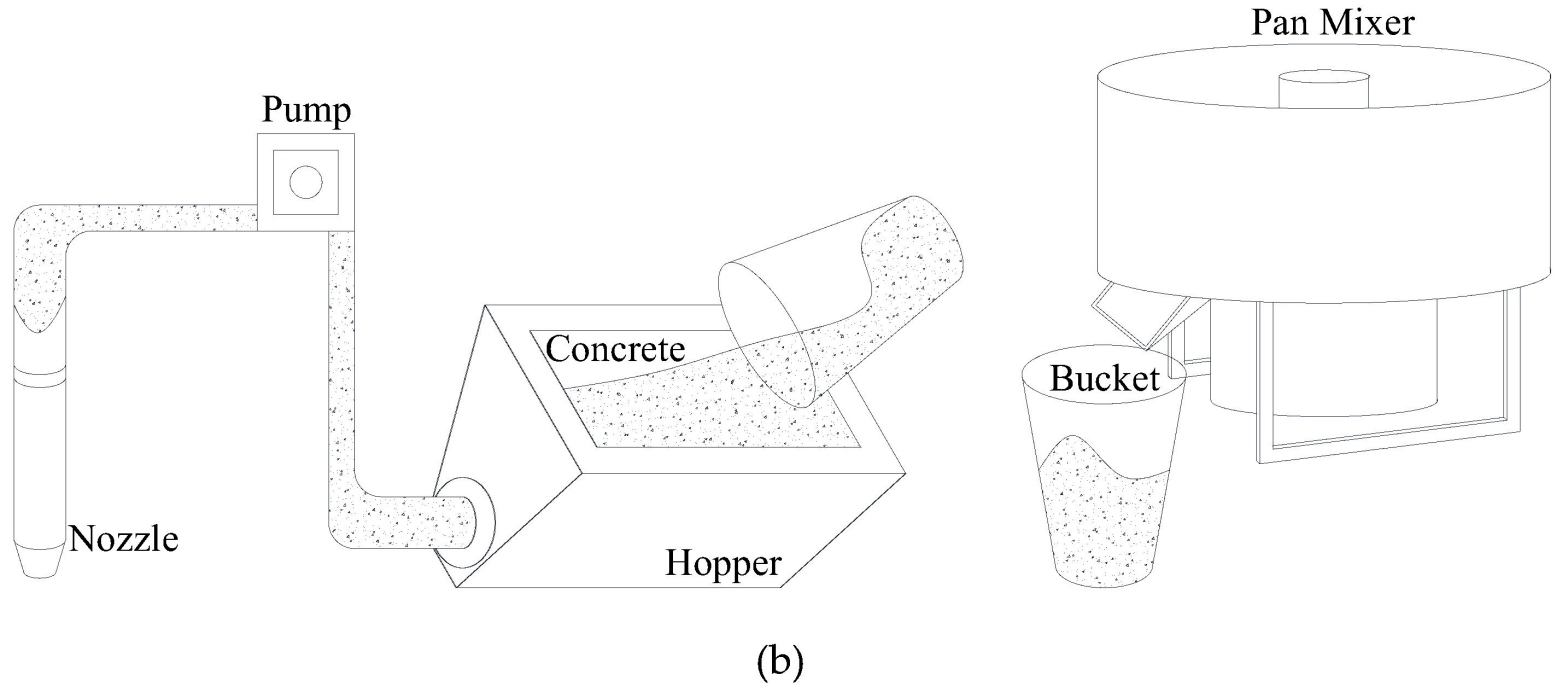
Classification of concrete supplying method. (**a**) Continuous supply; (**b**) Discontinuous supply.

The yield stress of fresh concrete increases with the increasing elapsed time and thus the time at which fresh concrete is supplied affects buildability. Figure 2a shows an ideal continuous supply method, in which a continuous mixer supplies a fixed amount of fresh concrete per second required for printing. In this case, fresh concrete remains in the hopper for a very short time. On the other hand, the example shown in Figure 2b is of a discontinuous supply method in which fresh concrete has a certain amount of elapsed time before printing as it is supplied only when the hopper is completely emptied. In this case, fresh concrete is kept in the hopper for a relatively long time compared to a continuous supply method. A typical concrete mixer adopts the same method as that of the pan mixer shown in Figure 2b. Therefore, this study sought to derive the initial yield stress and hardening coefficient required for the build-up layers of fresh concrete under the assumption of the discontinuous supply shown in Figure 2b. In addition, the following assumptions were made to simplify the calculation of the maximum shear stress in fresh concrete.

**Figure 3:**
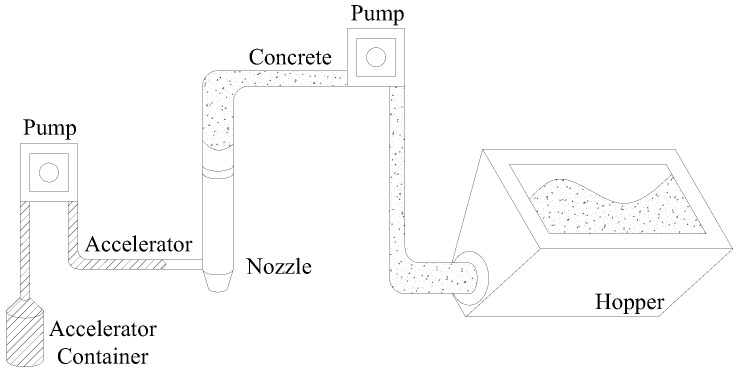
Schematic diagram of concrete printer.

**Figure 4:**
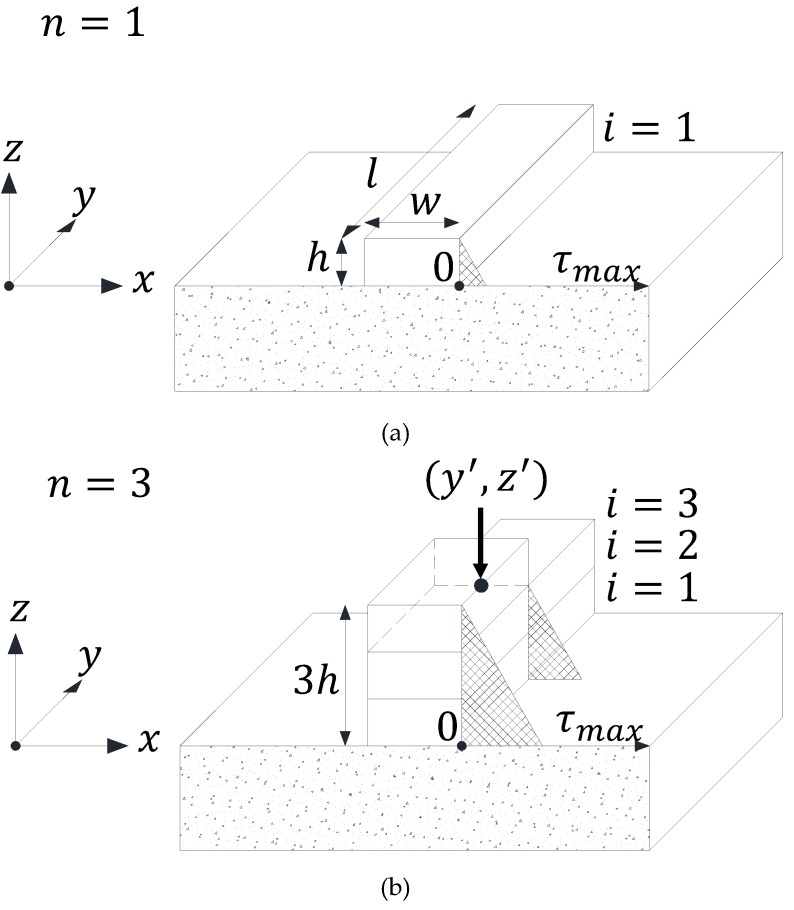
Maximum shear stress distribution of built up fresh concrete. (**a**) Case of n=1 & fully built up layer; (**b**) Case of n=3 & half laminated layer.

1. The accelerator is injected from the nozzle, as shown in Figure 3 and a fixed quantity is injected in proportion to the unit discharge amount.
2. The cross section of fresh concrete is a perfect rectangle, as shown in Figure 4.
3. Fresh concrete is built up vertically and the width (w), height (h) and length (l) of each layer of printed concrete are the same.

## 3. Proposed Model

### 3.1. Maximum Shear Stress Distribution (Demand Curve)

In Figure 4, *n* represents the total number of built up layers and *i* means the order of the layers built up from the bottom surface. Normal compressive stress (σ) generated from any height *z* to an *xy* plane can be calculated as shown below [15,16,17,18].

(1) $$\sigma (z)\;=\;\rho g\frac{wl(nh-z)}{wl}=\rho g(nh-z)$$

where *ρ* is the density of fresh concrete and *g* is the gravity acceleration. The maximum shear stress (*τ*_max_ ) that occurs in the layered fresh concrete can be calculated from the equilibrium condition of forces, as shown below [15,16,17,18].

(2) $$\tau_{\max}(z)=\frac{1}{2}\sigma (z)=\frac{1}{2}\rho g(nh-z)$$

Figure 4a shows the maximum shear stress distribution according to Equation (2). On the other hand, Figure 4b shows the maximum shear stress distribution when fresh concrete is not fully formed but partially formed within the same layer. In that case, the normal compressive stress and maximum shear stress generated from the position (y′,z′) of a build-up layer point to the *xy* plane can be calculated as shown below.

(3) $$\sigma (y,z)=\{\begin{array}{cc} \rho g(z\prime +h-z) & \left(\mathrm{where},\;0\leq y\leq y\prime ,\;0\leq z\leq z\prime +h\right)\\ \rho g(z\prime -z) & \left(\mathrm{where},\;y\prime <y\leq l,\;0\leq z\leq z\prime \right)\\ 0 & \left(\mathrm{where},\;y\prime <y\leq l,\;z\prime <z\leq z\prime +h\right) \end{array}$$

(4) $$\tau_{\max}(y,z)=\{\begin{array}{cc} \frac{1}{2}\rho g(z\prime +h-z) & \left(\mathrm{where},\;0\leq y\leq y\prime ,\;0\leq z\leq z\prime +h\right)\\ \frac{1}{2}\rho g(z\prime -z) & \left(\mathrm{where},\;y\prime <y\leq l,\;0\leq z\leq z\prime \right)\\ 0 & \left(\mathrm{where},\;y\prime <y\leq l,\;z\prime <z\leq z\prime +h\right) \end{array}$$

In addition to the self-weight of fresh concrete, considering the extra shear stress ($\tau_{ex}$) caused by discharge pressure at the point where the printing is carried out, if any, the maximum shear stress ($\tau_{\max}$) in the layered fresh concrete can be re-expressed, as follows.

(5) $$\tau_{\max}(y,z)=\{\begin{array}{cc} \frac{1}{2}\rho g(z\prime +h-z) & \left(where,0\leq y<y\prime ,0\leq z\leq z\prime +h\right)\\ \frac{1}{2}\rho g(z\prime +h-z)+\tau_{ex} & \left(where,y=y\prime ,0\leq z\leq z\prime +h\right)\\ \frac{1}{2}\rho g(z\prime -z) & \left(where,y\prime <y\leq l,0\leq z\leq z\prime \right)\\ 0 & \left(where,y\prime <y\leq l,z\prime <z\leq z\prime +h\right) \end{array}$$

### 3.2. Elapsed Time

The yield stress distribution of fresh concrete varies from hour to hour, even during the printing process, because the yield stress of fresh concrete changes with the elapsed time. The elapsed time can be calculated by dividing the length of path by the velocity of nozzle movement.

**Figure 5:**
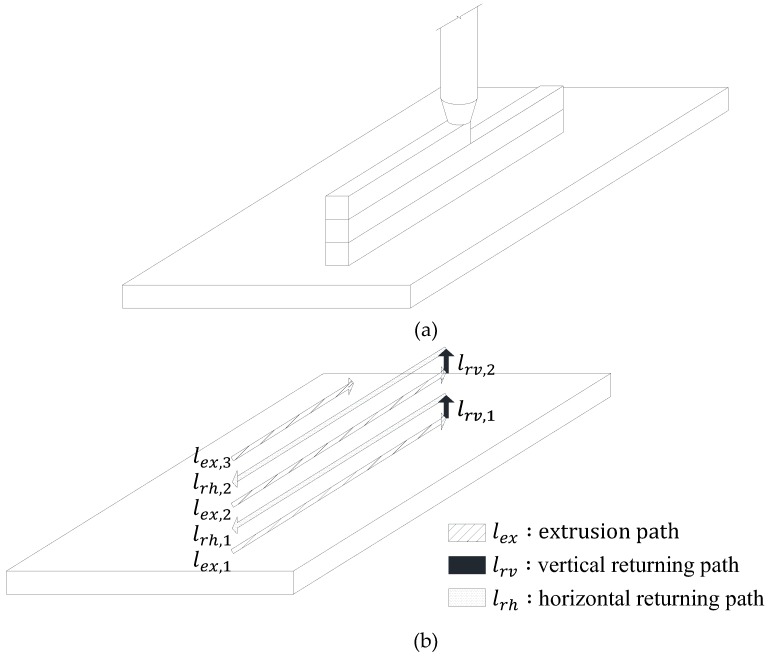

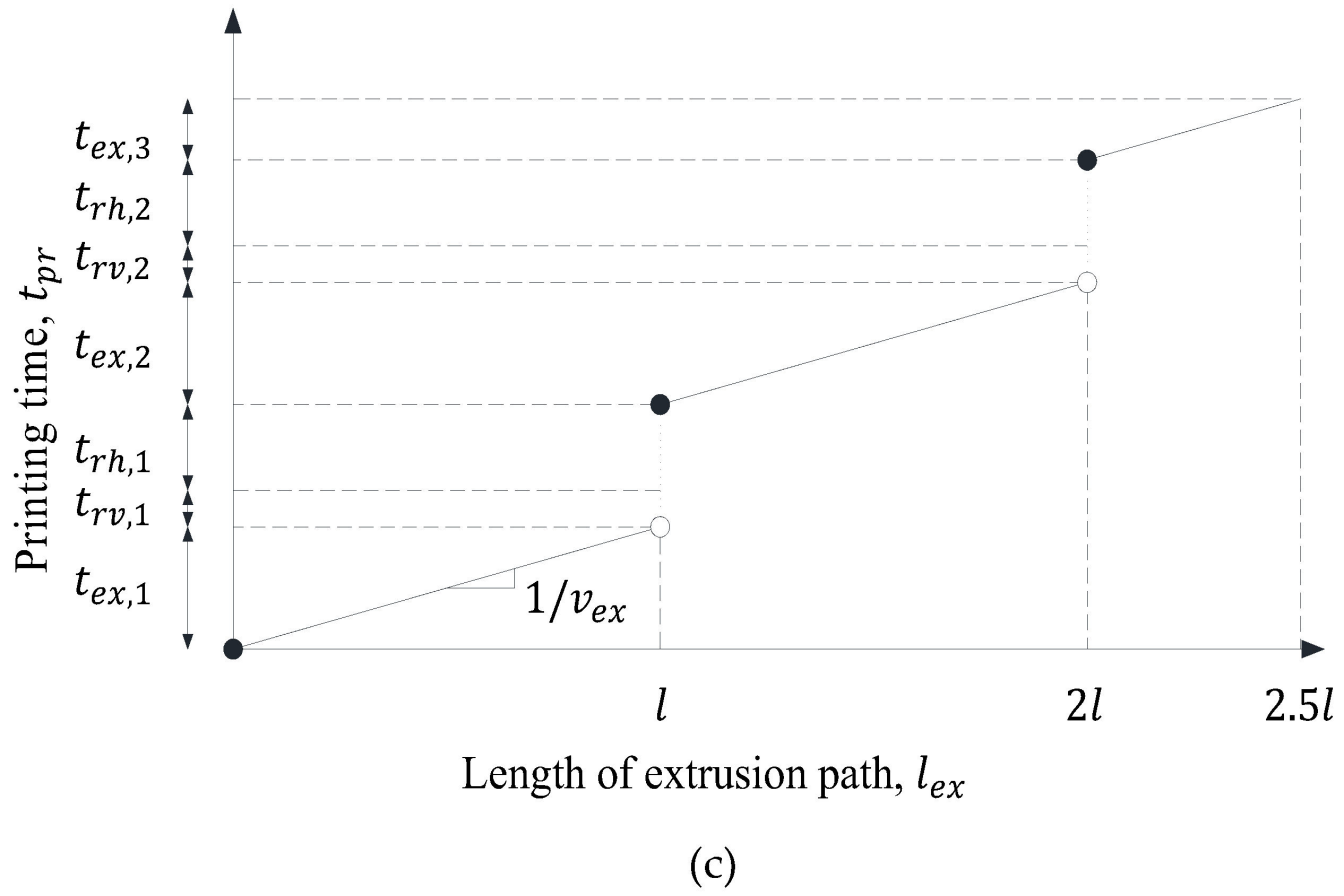
Classification of printing path ($l_{pr}$) & $l_{ex}-t_{pr}$ curve. (**a**) Vertically layered fresh concrete; (**b**) Classification of printing path ($l_{pr}$); (**c**) $l_{ex}-t_{pr}$ curve.

As shown in Figure 5a, if fresh concrete is vertically built up with the same width, length and height in each layer, the length of the printing path ($l_{pr,i}$) from the $i^{th}$ layer to arbitrary time (t) is classified into the length of the extrusion path ($l_{ex,i}$), the length of the horizontal returning path ($l_{rh,i}$) and the length of vertical returning path ($l_{rv,i}$), as shown in Figure 5b and this can be represented by the equation below.

(6) $$l_{pr,i}=l_{ex,i}+l_{rh,i}+l_{rv,i}$$

Therefore, the total length ($l_{ex}$,$l_{rh}$,$l_{rv}$) considering the total printing process and the length of the total printing path ($l_{pr}$) can be calculated by summing up the printing paths at each layer, as shown below.

(7a) $$l_{ex}=\sum l_{ex,i}$$

(7b) $$l_{rh}=\sum l_{rh,i}$$

(7c) $$l_{rv}=\sum l_{rv,i}$$

(7d) $$l_{pr}=\sum l_{pr,i}$$

If the velocity of nozzle movement along each path is $v_{ex}$,$v_{rh}$, $v_{rv}$, the time($t_{ex,i}$,$t_{rh,i}$$t_{rv,i}$) required to move along each path at the $i^{th}$ layer is shown below.

(8a) $$t_{ex,i}=\frac{l_{ex,i}}{v_{ex}}$$

(8b) $$t_{rh,i}=\frac{l_{rh,i}}{v_{rh}}$$

(8c) $$t_{rv,i}=\frac{l_{rv,i}}{v_{rv}}$$

Therefore, the time ($t_{pr,i}$) required for printing the $i^{th}$ layer is the sum of $t_{ex,i}$, $t_{rh,i}$, $t_{rv,i}$ and this can be represented by the equation below.

(9) $$t_{pr,i}=t_{ex,i}+t_{rh,i}+t_{rv,i}$$

In the same way, the total time taken to move along each path ($t_{ex}$, $t_{rh}$, $t_{rv}$) and the total time spent on the total printing process ($t_{pr}$) can be calculated as follows.

(10a) $$t_{ex}=\sum t_{ex,i}$$

(10b) $$t_{rh}=\sum t_{rh,i}$$

(10c) $$t_{rv}=\sum t_{rv,i}$$

(10d) $$t_{pr}=\sum t_{pr,i}$$

On the other hand, since fresh concrete is formed only in the extrusion path during the printing process, the relationship between $l_{ex}$ and $t_{pr}$ is represented by a discontinuous function, as shown in Figure 5c and this can be represented using the floor function, as follows.

(11) $$t_{pr}(l_{ex})=(\frac{l}{v_{rh}}+\frac{h}{v_{rv}})\left\lfloor \frac{l_{ex}}{l}\right\rfloor +\frac{l_{ex}}{v_{ex}}$$

**Figure 6:**
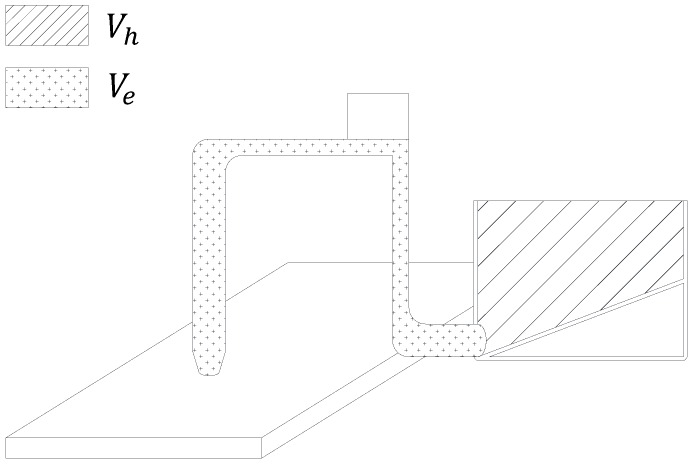
Volume of supplied fresh concrete into hopper.

Figure 6 shows the volume of fresh concrete supplied to the hopper and pipe. In this study, it was assumed that the amount of fresh concrete as much as the sum of the volume of the empty space ($V_{e}$) from the start point of the pipe to the end point of the nozzle and the volume of the hopper ($V_{h}$) is first supplied and then fresh concrete is supplied as much as $V_{h}$. The length of the filament ($l_{s}$) that can be printed by fresh concrete as much as $V_{h}$ can be calculated as shown below.

(12) $$l_{s}=\frac{V_{h}}{wh}$$

Where w and h are the width and height of the filament, respectively. If the number of times for concrete supply is m, fresh concrete has to be supplied at a point in time $l_{ex}=(m-1)l_{s}$. In this case, the mth fresh concrete supply point $t_{s,m}$ can be calculated by substituting $(m-1)l_{s}$ into Equation (11), as shown below.

(13) $$t_{s,m}=t_{pr}((m-1)l_{s})=(\frac{l}{v_{rh}}+\frac{h}{v_{rv}})\left\lfloor \frac{(m-1)l_{s}}{l}\right\rfloor +\frac{(m-1)l_{s}}{v_{ex}}$$

**Figure 7:**
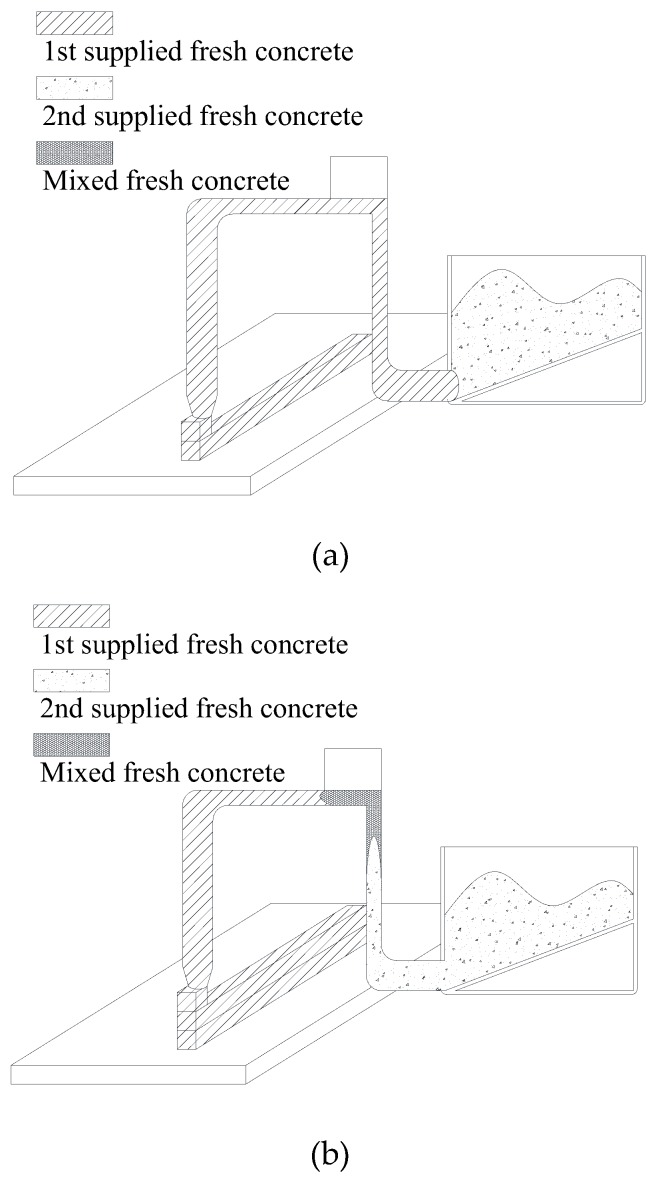

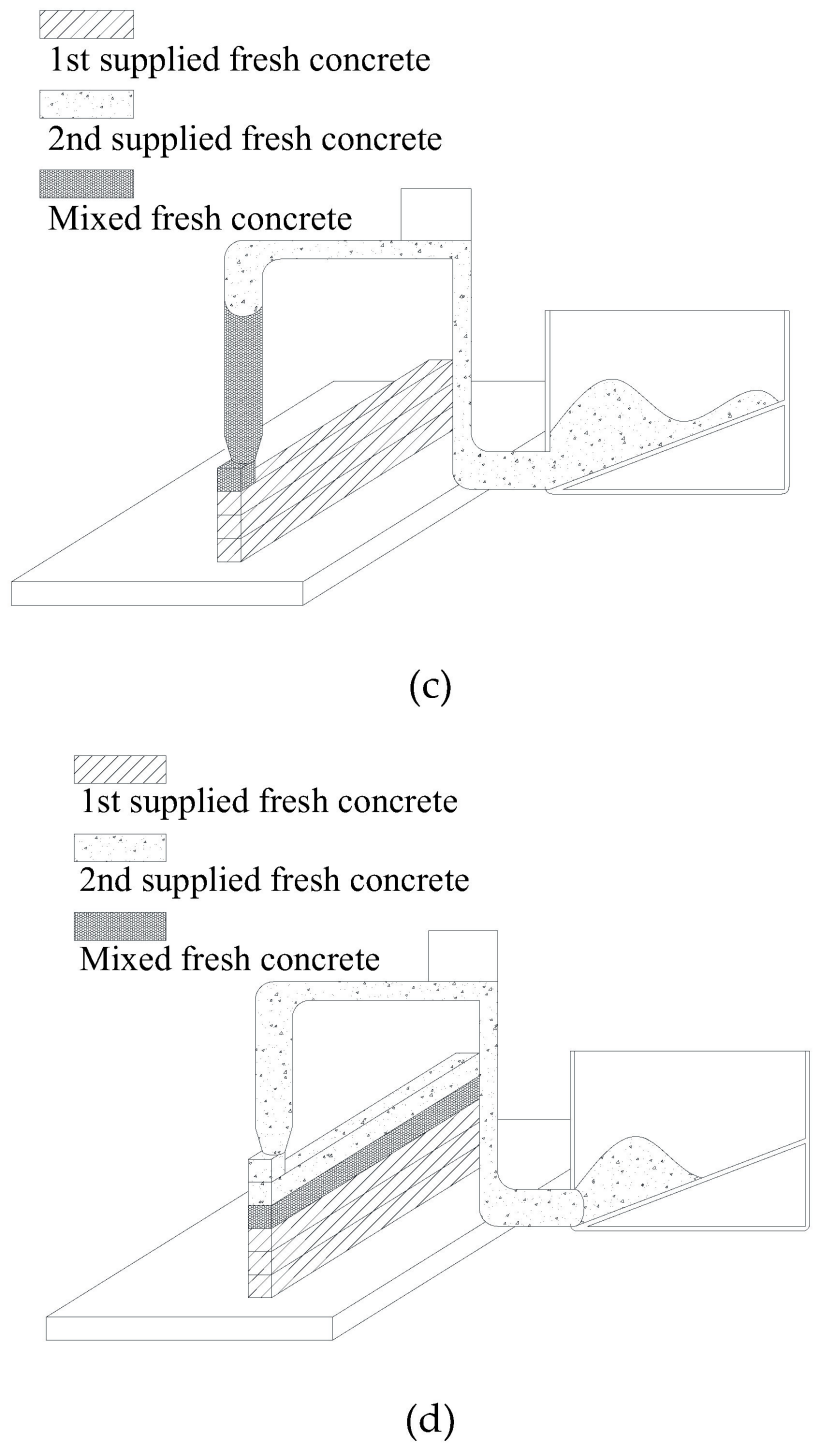
Extrusion phase of fresh concrete. (**a**) 1st supplied fresh concrete extrusion phase; (**b**) Fresh concrete mixing phase; (**c**) Mixed fresh concrete extrusion phase; (**d**) 2nd supplied fresh concrete extrusion phase.

Figure 7 shows the extrusion phase of fresh concrete. At the beginning of printing, the 1st-supplied fresh concrete contained in the pipe is extruded as shown in Figure 7a. After that, the 1st-supplied fresh concrete and the 2nd-supplied fresh concrete begin to be mixed in the pipe as shown in Figure 7b and then the mixed fresh concrete is extruded as shown in Figure 7c. After this step, the 2nd-supplied fresh concrete is extruded as shown in Figure 7d. Consequently, it is very difficult to predict the yield stress of the mixed fresh concrete in this multiphase flow process. Therefore, to calculate the yield stress on safe side and make it simple, this study set the elapsed time of fresh concrete to be zero when the 2nd fresh concrete is supplied into the hopper, although the remaining fresh concrete in the pipe is the 1st-supplied fresh concrete or mixed fresh concrete.

The elapsed time ($t_{e}$) experienced by fresh concrete is the time elapsed from the mixture of concrete to the moment of printing. Thus, it can be calculated by the difference between $t_{pr}$ and $t_{s,m}$, as shown below.

(14) $$t_{e}=t_{pr}-t_{s,m}$$

In the study conducted by Le et al. [6,7], an accelerator was added to rapidly increase the yield stress of the fresh concrete. Since the accelerator is injected in the vicinity of the nozzle, the velocity of concrete hardening increases from the time when concrete is extruded. Therefore, the relationship between elapsed time and the yield stress of fresh concrete changes from the point in time of the extrusion and the total elapsed time ($t_{e}$) experienced by fresh concrete can be classified into the elapsed time experienced before extrusion($t_{b}$) and the elapsed time ($t_{a}$) experienced after extrusion.

(15) $$t_{e}=t_{b}+t_{a}$$

If the length of the extrusion path for concrete present at an arbitrary location to be printed is $l_{o}$ and the time when concrete located at $l_{o}$ is extruded is $t_{o}$, the $t_{o}$ can be calculated by substituting the $l_{o}$ into $t_{pr}(l_{ex})$ function, as shown below.

(16) $$t_{o}=t_{pr}(l_{o})=(\frac{l}{v_{rh}}+\frac{h}{v_{rv}})\left\lfloor \frac{l_{o}}{l}\right\rfloor +\frac{l_{o}}{v_{ex}}$$

When the fresh concrete located at $l_{o}$ is extruded, the number of times concrete must be supplied can be calculated using the floor function, as follows.

(17) $$m=\left\lfloor \frac{l_{o}}{l_{s}}+1\right\rfloor$$

The $t_{s,m}$ of concrete located at $l_{o}$ can be estimated by substituting m calculated from Equation (17) into Equation (13). Since $t_{b}$ is the elapsed time experienced from the mixture of concrete to the extrusion of concrete, it is the difference between $t_{o}$ and $t_{s,m}$. Likewise, as $t_{a}$ is the elapsed time experienced from the extrusion of concrete to the moment of printing, it is the difference between $t_{pr}$ and $t_{o}$. Therefore, $t_{b}$ and $t_{a}$ can be represented by the following equations, respectively.

(18a) $$t_{b}=t_{o}-t_{s,m}$$

(18b) $$t_{a}=t_{pr}-t_{o}$$

**Figure 8:**
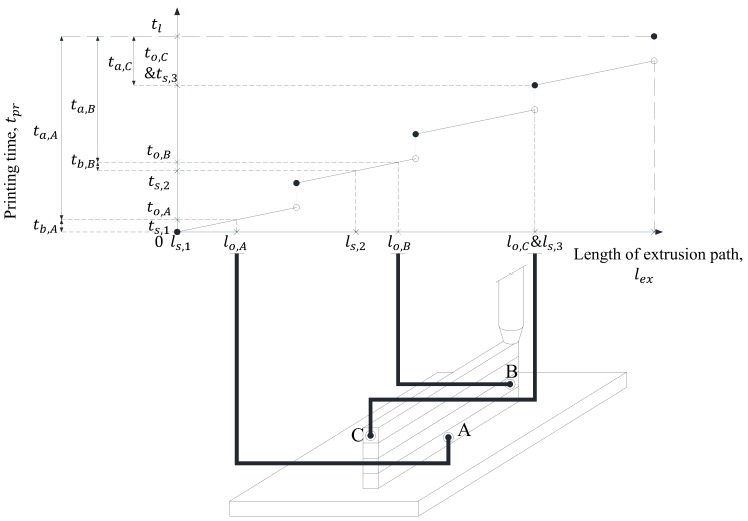
Calculation procedure of elapsed time according to element position.

Figure 8 shows the elapsed time $t_{b}$, $t_{a}$ calculation procedure of concrete located at points A, B and C in the printing completion stage during the printing process of building up four layers. For example, since concrete at point A is located in $l_{o,A}$, the time when printed is $t_{o,A}$ and concrete located at A is supplied from $t_{s,1}$, the equation becomes $t_{b,A}=t_{o,A}-t_{s,1}$. In addition, since the printing end point is $t_{pr}(4l)=t_{l}$, it is $t_{a,A}=t_{l}-t_{o,A}$.

### 3.3. Relationship between Elapsed Time and Yield Stress

To define failure criteria, Wolfs et al. [26] used Mohr-Coulomb criterion, while Murata [15], Christensen [16], Pashias et al. [17] and Saak et al. [18] used Tresca criterion, in which the yield stress of fresh concrete was obtained from rheometer measurements. They reported that both Tresca and Mohr-Coulomb criteria well predicted the failure of fresh concrete and thus this study adopted Tresca criterion that is simpler than Mohr-Coulomb criterion.

Previous research [19,20,21] found that a liner or exponential function can be used to approximate the increase in the yield stress of fresh concrete according to the elapsed time. That is, the elapsed time-yield stress equations can be represented as shown below.

(19a) $$\tau_{y}(t_{e})=\tau_{0}e^{\alpha t_{e}}$$

(19b) $$\tau_{y}(t_{e})=\alpha t_{e}+\tau_{0}$$

Where α and $\tau_{0}$ are the hardening coefficient and the initial yield stress when the accelerator is not used. Equation (19a) or Equation (19b) can be selected and applied based on the yield stress measurement results of fresh concrete. However, the yield stress derived from the elapsed time-yield stress equation should be always smaller than the measured value in order to obtain safe results. It is noted that yield stress of fresh concrete should be measured after discharge because the yield stress can be changed due to the energy introduced into the concrete during the pumping and transport processes.

If the accelerator is injected into fresh concrete, the yield stress of the fresh concrete increases more rapidly from the elapsed time $t_{b}$ of the accelerator injection. To take this phenomenon into consideration, a hardening coefficient β was additionally introduced in this study, where β is a constant that can be obtained by measuring the yield stress of fresh concrete according to the elapsed time after the accelerator is injected into the fresh concrete. The elapsed time-yield stress equation can be represented as shown below.

(20a) $$\tau_{y}(t_{b},t_{a})=\tau_{0}e^{\alpha t_{b}+\beta (t_{e}-t_{b})}=\tau_{0}e^{\alpha t_{b}+\beta t_{a}}$$

(20b) $$\tau_{y}(t_{b},t_{a})=\alpha t_{b}+\beta (t_{e}-t_{b})+\tau_{0}=\alpha t_{b}+\beta t_{a}+\tau_{0}$$

### 3.4. Yield Stress Distribution (Capacity Curve) 

If the accelerator is not injected into the fresh concrete, the yield stress according to the position of the fresh concrete ($l_{o}$) and the position where the fresh concrete is extruded ($l_{ex}$) can be calculated using Equations (11), (13), (14), (17), (19a) and (19b), as shown below.

(21a) $$\tau_{y}(l_{o},l_{ex})=\tau_{0}e^{\alpha ((\frac{l}{v_{rh}}+\frac{h}{v_{rv}})(\left\lfloor \frac{l_{ex}}{l}\right\rfloor -\left\lfloor \frac{(\left\lfloor (l_{o}+l_{s})/l_{s}\right\rfloor -1)l_{s}}{l}\right\rfloor )+\frac{l_{ex}-(\left\lfloor (l_{o}+l_{s})/l_{s}\right\rfloor -1)l_{s}}{v_{ex}})}$$

(21b) $$\tau_{y}(l_{o},l_{ex})=\alpha ((\frac{l}{v_{rh}}+\frac{h}{v_{rv}})(\left\lfloor \frac{l_{ex}}{l}\right\rfloor -\left\lfloor \frac{(\left\lfloor (l_{o}+l_{s})/l_{s}\right\rfloor -1)l_{s}}{l}\right\rfloor )+\frac{l_{ex}-(\left\lfloor (l_{o}+l_{s})/l_{s}\right\rfloor -1)l_{s}}{v_{ex}})+\tau_{0}$$

If the accelerator is injected into the fresh concrete, the yield stress according to the position of the fresh concrete ($l_{o}$) and the position where the fresh concrete is extruded ($l_{ex}$) can be calculated using Equations (11), (13), (16), (17), (18a), (18b), (20a) and (20b), as shown below.

(22a) $$\tau_{y}(l_{o},l_{ex})=\tau_{0}e^{\alpha ((\frac{l}{v_{rh}}+\frac{h}{v_{rv}})(\left\lfloor \frac{l_{o}}{l}\right\rfloor -\left\lfloor \frac{(\left\lfloor (l_{o}+l_{s})/l_{s}\right\rfloor -1)l_{s}}{l}\right\rfloor )+\frac{l_{o}-(\left\lfloor (l_{o}+l_{s})/l_{s}\right\rfloor -1)l_{s}}{v_{ex}})+\beta ((\frac{l}{v_{rh}}+\frac{h}{v_{rv}})(\left\lfloor \frac{l_{ex}}{l}\right\rfloor -\left\lfloor \frac{l_{o}}{l}\right\rfloor )+\frac{l_{ex}-l_{o}}{v_{ex}})}$$

(22b) $$\begin{array}{l} \tau_{y}(l_{o},l_{ex})=\alpha ((\frac{l}{v_{rh}}+\frac{h}{v_{rv}})(\left\lfloor \frac{l_{o}}{l}\right\rfloor -\left\lfloor \frac{(\left\lfloor (l_{o}+l_{s})/l_{s}\right\rfloor -1)l_{s}}{l}\right\rfloor )+\frac{l_{o}-(\left\lfloor (l_{o}+l_{s})/l_{s}\right\rfloor -1)l_{s}}{v_{ex}})\\ \;\;\;\;\;\;\;\;\;\;\;\;\;\;\;\;\;+\beta ((\frac{l}{v_{rh}}+\frac{h}{v_{rv}})(\left\lfloor \frac{l_{ex}}{l}\right\rfloor -\left\lfloor \frac{l_{o}}{l}\right\rfloor )+\frac{l_{ex}-l_{o}}{v_{ex}})+\tau_{0} \end{array}$$

The yield stress calculated using Equation (22a,b) are a function with respect to $l_{o}$, $l_{ex}$ and the yield stresses of Equation (22a,b) should be represented as a function of (y,z) for comparison with the maximum shear stress ($\tau_{\max}$) represented by Equation (5). In addition, since the maximum shear stress is largest at the bottom of each layer, the yield stresses for each layer should be compared at that same location. The following transformation equations can be used to represent $l_{o}$ and $l_{ex}$ as a function of (y,z) and a function of (y′,z′) at the bottom of each layer.

(23a) $$l_{o}(y,z)=y+l\left\lfloor z/h\right\rfloor$$

(23b) $$l_{ex}(y\prime ,z\prime )=y\prime +l\left\lfloor z\prime /h\right\rfloor$$

If Equation (23a,b) is substituted into Equations (21a), (21b), (22a) and (22b), the yield stress $\tau_{y}(y,z,y\prime ,z\prime )$ can be represented by the following equations.

(24a) $$\tau_{y}(y,z,y\prime ,z\prime )=\{\begin{array}{l} \tau_{0}e^{\alpha ((\frac{l}{v_{rh}}+\frac{h}{v_{rv}})(\left\lfloor \frac{y\prime +l\left\lfloor z\prime /h\right\rfloor }{l}\right\rfloor -\left\lfloor \frac{(\left\lfloor (y+l\left\lfloor z/h\right\rfloor +l_{s})/l_{s}\right\rfloor -1)l_{s}}{l}\right\rfloor )+\frac{y\prime +l\left\lfloor z\prime /h\right\rfloor -(\left\lfloor (y+l\left\lfloor z/h\right\rfloor +l_{s})/l_{s}\right\rfloor -1)l_{s}}{v_{ex}})}\\ \;\;\;\;\;\;\;\;\;\;\;\;\;\;\;\;\;\;\;\;\;\;\;\;\;\;\;\;\;\;\;\;(where,\;\;\;0\leq y\leq y\prime ,0\leq z<z\prime +h\;\;or\;\;y\prime <y\leq l,0\leq z<z\prime )\\ \;\;\;\;\;\;\;\;\;\;\;\;\;\;\;0\;\;\;\;\;\;\;\;\;\;\;\;\;\;\;(where,\;\;0\leq y\leq y\prime ,z=z\prime +h\;\;or\;\;y\prime <y\leq l,z\prime \leq z\leq z\prime +h) \end{array}$$

(24b) $$\tau_{y}(y,z,y\prime ,z\prime )=\{\begin{array}{l} \alpha (\frac{l}{v_{rh}}+\frac{h}{v_{rv}})(\left\lfloor \frac{y\prime +l\left\lfloor z\prime /h\right\rfloor }{l}\right\rfloor -\left\lfloor \frac{(\left\lfloor (y+l\left\lfloor z/h\right\rfloor +l_{s})/l_{s}\right\rfloor -1)l_{s}}{l}\right\rfloor )\\ \;+\alpha (\frac{y\prime +l\left\lfloor z\prime /h\right\rfloor -(\left\lfloor (y+l\left\lfloor z/h\right\rfloor +l_{s})/l_{s}\right\rfloor -1)l_{s}}{v_{ex}})+\tau_{0}\;\;\;\;\;\;\;\;\;\;\;\;\;\;\;\;\;\;\;\;\;\;\\ \;\;\;\;\;\;\;\;\;\;\;\;\;\;\;\;\;\;\;\;\;\;\;\;\;\;\;\;\;(where,0\leq y\leq y\prime ,0\leq z<z\prime +h\;\;or\;\;y\prime <y\leq l,0\leq z<z\prime )\\ 0\;\;\;\;\;\;\;\;\;\;\;\;\;\;\;\;\;\;\;\;\;\;\;\;\;\;\;(where,0\leq y\leq y\prime ,z=z\prime +h\;\;or\;\;y\prime <y\leq l,z\prime \leq z\leq z\prime +h) \end{array}$$

(24c) $$\tau_{y}(y,z,y\prime ,z\prime )=\{\begin{array}{l} \tau_{0}e^{\alpha ((\frac{l}{v_{rh}}+\frac{h}{v_{rv}})(\left\lfloor \frac{y+l\left\lfloor z/h\right\rfloor }{l}\right\rfloor -\left\lfloor \frac{(\left\lfloor (y+l\left\lfloor z/h\right\rfloor +l_{s})/l_{s}\right\rfloor -1)l_{s}}{l}\right\rfloor )+\frac{y+l\left\lfloor z/h\right\rfloor -(\left\lfloor (y+l\left\lfloor z/h\right\rfloor +l_{s})/l_{s}\right\rfloor -1)l_{s}}{v_{ex}})}\\ \times e^{\beta ((\frac{l}{v_{rh}}+\frac{h}{v_{rv}})(\left\lfloor \frac{y\prime +l\left\lfloor z\prime /h\right\rfloor }{l}\right\rfloor -\left\lfloor \frac{y+l\left\lfloor z/h\right\rfloor }{l}\right\rfloor )+\frac{y\prime +l\left\lfloor z\prime /h\right\rfloor -(y+l\left\lfloor z/h\right\rfloor )}{v_{ex}})\;\;\;\;\;\;\;\;\;\;\;\;\;\;\;\;\;\;\;\;\;\;\;\;\;\;\;\;\;\;\;\;\;\;\;\;\;\;\;\;\;\;\;\;\;\;\;\;\;\;\;\;\;\;\;\;\;\;\;\;\;\;\;\;\;\;\;\;\;\;\;\;\;}\\ \;\;\;\;\;\;\;\;\;\;\;\;\;\;\;\;\;\;\;\;\;\;\;\;\;\;\;\;\;\;\;\;\;\;\;\;\;\;\;\;\;\;\;\;(where,0\leq y\leq y\prime ,0\leq z<z\prime +h\;\;or\;\;y\prime <y\leq l,0\leq z<z\prime )\\ \;\;\;\;\;\;\;\;\;\;\;\;\;\;0\;\;\;\;\;\;\;\;\;\;\;\;\;\;\;\;\;\;\;\;\;\;\;\;\;\;\;(where,0\leq y\leq y\prime ,z=z\prime +h\;\;or\;\;y\prime <y\leq l,z\prime \leq z\leq z\prime +h) \end{array}$$

(24d) $$\tau_{y}(y,z,y\prime ,z\prime )=\{\begin{array}{l} \alpha (\frac{l}{v_{rh}}+\frac{h}{v_{rv}})(\left\lfloor \frac{y+l\left\lfloor z/h\right\rfloor }{l}\right\rfloor -\left\lfloor \frac{(\left\lfloor (y+l\left\lfloor z/h\right\rfloor +l_{s})/l_{s}\right\rfloor -1)l_{s}}{l}\right\rfloor )\\ +\alpha (\frac{y+l\left\lfloor z/h\right\rfloor -(\left\lfloor (y+l\left\lfloor z/h\right\rfloor +l_{s})/l_{s}\right\rfloor -1)l_{s}}{v_{ex}})\;\;\;\;\;\;\;\;\;\;\;\;\;\;\;\;\;\;\;\;\;\;\;\;\;\;\;\;\;\;\;\;\;\\ +\beta (\frac{l}{v_{rh}}+\frac{h}{v_{rv}})(\left\lfloor \frac{y\prime +l\left\lfloor z\prime /h\right\rfloor }{l}\right\rfloor -\left\lfloor \frac{y+l\left\lfloor z/h\right\rfloor }{l}\right\rfloor )\;\;\;\;\;\;\;\;\;\;\;\;\;\;\;\;\;\;\;\;\;\;\;\;\;\;\;\;\;\;\;\\ +\beta (\frac{y\prime +l\left\lfloor z\prime /h\right\rfloor -(y+l\left\lfloor z/h\right\rfloor )}{v_{ex}})+\tau_{0}\;\;\;\;\;\;\;\;\;\;\;\;\;\;\;\;\;\;\;\;\;\;\;\;\;\;\;\;\;\;\;\;\;\;\;\;\;\;\;\;\;\;\;\;\;\;\;\;\;\;\;\;\;\;\\ \;\;\;\;\;\;\;\;\;\;\;\;\;\;\;\;\;\;\;\;\;\;\;\;\;\;\;\;\;(where,0\leq y\leq y\prime ,0\leq z<z\prime +h\;\;or\;\;y\prime <y\leq l,0\leq z<z\prime )\\ 0\;\;\;\;\;\;\;\;\;\;\;\;\;\;\;\;\;\;\;\;\;\;\;\;\;\;\;(where,0\leq y\leq y\prime ,z=z\prime +h\;\;or\;\;y\prime <y\leq l,z\prime \leq z\leq z\prime +h) \end{array}$$

That is, the yield stress $\tau_{y}(y,z,y\prime ,z\prime )$ at all positions on the y−z coordinate plane can be calculated using Equation (24a–d).

### 3.5. Demand $\tau_{o}$, α and β

Deformation occurs when the maximum shear stress generated in layered fresh concrete exceeds the yield stress [14,15,16,17,18]. To prevent this, the yield stress of the fresh concrete should be greater than the maximum shear stress to satisfy the following relation.

(25) $$\tau_{y}\geq \tau_{\max}$$

Therefore, the yield stress ($\tau_{0}$) required to build up one layer of fresh concrete at the beginning of printing without deformations should be satisfied in accordance with the following equation.

(26) $$\tau_{0}\geq \tau_{\max}=\frac{1}{2}(\rho gh)+\tau_{ex}$$

If the accelerator is not injected into fresh concrete, the α required to prevent deformation that may occur in the build-up process of fresh concrete can be calculated using Equations (5) and (24a), (24b) and (25).

(27a) $$\alpha (y,z,y\prime ,z\prime )\geq \{\begin{array}{ll} & \frac{ln(\frac{1}{2\tau_{0}}\rho g(z\prime +h-z))}{(\frac{l}{v_{rh}}+\frac{h}{v_{rv}})(\left\lfloor \frac{y\prime +l\left\lfloor z\prime /h\right\rfloor }{l}\right\rfloor -\left\lfloor \frac{(\left\lfloor (y+l\left\lfloor z/h\right\rfloor +l_{s})/l_{s}\right\rfloor -1)l_{s}}{l}\right\rfloor )+\frac{y\prime +l\left\lfloor z\prime /h\right\rfloor -(\left\lfloor (y+l\left\lfloor z/h\right\rfloor +l_{s})/l_{s}\right\rfloor -1)l_{s}}{v_{ex}}}\\ & \;\;\;\;\;\;\;\;\;\;\;\;\;\;\;\;\;\;\;\;\;\;\;\;\;\;\;\;\;\;\;\;\;\;\;\;\;\;\;\;\;\;\;\;\;\;\;\;\;\;\;\;\;\;\;\;\;\;(where,0\leq y<y\prime ,0\leq z<z\prime +h)\\ & \frac{ln(\frac{1}{2\tau_{0}}(\rho g(z\prime +h-z))+\frac{\tau_{ex}}{\tau_{0}})}{(\frac{l}{v_{rh}}+\frac{h}{v_{rv}})(\left\lfloor \frac{y\prime +l\left\lfloor z\prime /h\right\rfloor }{l}\right\rfloor -\left\lfloor \frac{(\left\lfloor (y+l\left\lfloor z/h\right\rfloor +l_{s})/l_{s}\right\rfloor -1)l_{s}}{l}\right\rfloor )+\frac{y\prime +l\left\lfloor z\prime /h\right\rfloor -(\left\lfloor (y+l\left\lfloor z/h\right\rfloor +l_{s})/l_{s}\right\rfloor -1)l_{s}}{v_{ex}}}\\ & \;\;\;\;\;\;\;\;\;\;\;\;\;\;\;\;\;\;\;\;\;\;\;\;\;\;\;\;\;\;\;\;\;\;\;\;\;\;\;\;\;\;\;\;\;\;\;\;\;\;\;\;\;\;\;\;\;\;\;\;\;\;\;\;(where,y=y\prime ,0\leq z<z\prime +h)\\ & \frac{ln(\frac{1}{2\tau_{0}}\rho g(z\prime -z))}{(\frac{l}{v_{rh}}+\frac{h}{v_{rv}})(\left\lfloor \frac{y\prime +l\left\lfloor z\prime /h\right\rfloor }{l}\right\rfloor -\left\lfloor \frac{(\left\lfloor (y+l\left\lfloor z/h\right\rfloor +l_{s})/l_{s}\right\rfloor -1)l_{s}}{l}\right\rfloor )+\frac{y\prime +l\left\lfloor z\prime /h\right\rfloor -(\left\lfloor (y+l\left\lfloor z/h\right\rfloor +l_{s})/l_{s}\right\rfloor -1)l_{s}}{v_{ex}}}\\ & \;\;\;\;\;\;\;\;\;\;\;\;\;\;\;\;\;\;\;\;\;\;\;\;\;\;\;\;\;\;\;\;\;\;\;\;\;\;\;\;\;\;\;\;\;\;\;\;\;\;\;\;\;\;\;\;\;\;\;\;\;\;\;\;(where,y\prime <y\leq l,0\leq z<z\prime )\\ 0 & \;\;\;\;\;\;\;\;\;\;\;\;\;\;\;\;\;\;\;\;\;\;\;(where,0\leq y\leq y\prime ,z=z\prime +h\;\;or\;\;y\prime <y\leq l,z\prime \leq z\leq z\prime +h) \end{array}$$

(27b) $$\alpha (y,z,y\prime ,z\prime )\geq \{\begin{array}{c} \frac{\frac{1}{2}\rho g(z\prime +h-z)-\tau_{0}}{(\frac{l}{v_{rh}}+\frac{h}{v_{rv}})(\left\lfloor \frac{y\prime +l\left\lfloor z\prime /h\right\rfloor }{l}\right\rfloor -\left\lfloor \frac{(\left\lfloor (y+l\left\lfloor z/h\right\rfloor +l_{s})/l_{s}\right\rfloor -1)l_{s}}{l}\right\rfloor )+\frac{y\prime +l\left\lfloor z\prime /h\right\rfloor -(\left\lfloor (y+l\left\lfloor z/h\right\rfloor +l_{s})/l_{s}\right\rfloor -1)l_{s}}{v_{ex}}}\\ \;\;\;\;\;\;\;\;\;\;\;\;\;\;\;\;\;\;\;\;\;\;\;\;\;\;\;\;\;\;\;\;\;\;\;\;\;\;\;\;\;\;\;\;\;\;\;\;\;\;\;\;\;\;\;\;\;\;(where,0\leq y<y\prime ,0\leq z<z\prime +h)\\ \frac{\frac{1}{2}\rho g(z\prime +h-z)+\tau_{ex}-\tau_{0}}{(\frac{l}{v_{rh}}+\frac{h}{v_{rv}})(\left\lfloor \frac{y\prime +l\left\lfloor z\prime /h\right\rfloor }{l}\right\rfloor -\left\lfloor \frac{(\left\lfloor (y+l\left\lfloor z/h\right\rfloor +l_{s})/l_{s}\right\rfloor -1)l_{s}}{l}\right\rfloor )+\frac{y\prime +l\left\lfloor z\prime /h\right\rfloor -(\left\lfloor (y+l\left\lfloor z/h\right\rfloor +l_{s})/l_{s}\right\rfloor -1)l_{s}}{v_{ex}}}\\ \;\;\;\;\;\;\;\;\;\;\;\;\;\;\;\;\;\;\;\;\;\;\;\;\;\;\;\;\;\;\;\;\;\;\;\;\;\;\;\;\;\;\;\;\;\;\;\;\;\;\;\;\;\;\;\;\;\;\;\;\;\;\;\;(where,y=y\prime ,0\leq z<z\prime +h)\\ \frac{\frac{1}{2}\rho g(z\prime -z)-\tau_{0}}{(\frac{l}{v_{rh}}+\frac{h}{v_{rv}})(\left\lfloor \frac{y\prime +l\left\lfloor z\prime /h\right\rfloor }{l}\right\rfloor -\left\lfloor \frac{(\left\lfloor (y+l\left\lfloor z/h\right\rfloor +l_{s})/l_{s}\right\rfloor -1)l_{s}}{l}\right\rfloor )+\frac{y\prime +l\left\lfloor z\prime /h\right\rfloor -(\left\lfloor (y+l\left\lfloor z/h\right\rfloor +l_{s})/l_{s}\right\rfloor -1)l_{s}}{v_{ex}}}\\ \;\;\;\;\;\;\;\;\;\;\;\;\;\;\;\;\;\;\;\;\;\;\;\;\;\;\;\;\;\;\;\;\;\;\;\;\;\;\;\;\;\;\;\;\;\;\;\;\;\;\;\;\;\;\;\;\;\;\;\;\;\;\;\;(where,y\prime <y\leq l,0\leq z<z\prime )\\ 0\;\;\;\;\;\;\;\;\;\;\;\;\;\;\;\;\;\;\;\;\;\;\;(where,0\leq y\leq y\prime z=z\prime +h\;\;or\;\;y\prime <y\leq l,z\prime \leq z\leq z\prime +h) \end{array}$$

If the accelerator is injected into fresh concrete, the β required to prevent deformation that may occur in the build-up process of fresh concrete can be derived using Equations (5) and (24c), (24d) and (26).

(28a) $$\beta (y,z,y\prime ,z\prime )\geq \{\begin{array}{cc} & \frac{\ln(\frac{1}{2\tau_{0}}\rho g(z\prime +h-z))-\alpha ((\frac{l}{v_{rh}}+\frac{h}{v_{rv}})(\left\lfloor \frac{y+l\left\lfloor z/h\right\rfloor }{l}\right\rfloor -\left\lfloor \frac{(\left\lfloor (y+l\left\lfloor z/h\right\rfloor +l_{s})/l_{s}\right\rfloor -1)l_{s}}{l}\right\rfloor )+\frac{y+l\left\lfloor z/h\right\rfloor -(\left\lfloor (y+l\left\lfloor z/h\right\rfloor +l_{s})/l_{s}\right\rfloor -1)l_{s}}{v_{ex}})}{(\frac{l}{v_{rh}}+\frac{h}{v_{rv}})(\left\lfloor \frac{y\prime +l\left\lfloor z\prime /h\right\rfloor }{l}\right\rfloor -\left\lfloor \frac{y+l\left\lfloor z/h\right\rfloor }{l}\right\rfloor )+\frac{y\prime +l\left\lfloor z\prime /h\right\rfloor -(y+l\left\lfloor z/h\right\rfloor )}{v_{ex}}}\;\;\;\;\;\;\;\;\;\;\;\;\;\;\;\;\;\;\;\\ & \;\;\;\;\;\;\;\;\;\;\;\;\;\;\;\;\;\;\;\;\;\;\;\;\;\;\;\;\;\;\;\;\;\;\;\;\;\;\;\;\;\;\;\;\;\;\;\;\;\;\;\;\;\;\;\;\;\;(where,0\leq y<y\prime ,0\leq z<z\prime +h)\\ & \frac{ln(\frac{1}{2\tau_{0}}\rho g(z\prime +h-z)+\frac{\tau_{ex}}{\tau_{0}})-\alpha ((\frac{l}{v_{rh}}+\frac{h}{v_{rv}})(\left\lfloor \frac{y+l\left\lfloor z/h\right\rfloor }{l}\right\rfloor -\left\lfloor \frac{(\left\lfloor (y+l\left\lfloor z/h\right\rfloor +l_{s})/l_{s}\right\rfloor -1)l_{s}}{l}\right\rfloor )+\frac{y+l\left\lfloor z/h\right\rfloor -(\left\lfloor (y+l\left\lfloor z/h\right\rfloor +l_{s})/l_{s}\right\rfloor -1)l_{s}}{v_{ex}})}{(\frac{l}{v_{rh}}+\frac{h}{v_{rv}})(\left\lfloor \frac{y\prime +l\left\lfloor z\prime /h\right\rfloor }{l}\right\rfloor -\left\lfloor \frac{y+l\left\lfloor z/h\right\rfloor }{l}\right\rfloor )+\frac{y\prime +l\left\lfloor z\prime /h\right\rfloor -(y+l\left\lfloor z/h\right\rfloor )}{v_{ex}}}\\ & \;\;\;\;\;\;\;\;\;\;\;\;\;\;\;\;\;\;\;\;\;\;\;\;\;\;\;\;\;\;\;\;\;\;\;\;\;\;\;\;\;\;\;\;\;\;\;\;\;\;\;\;\;\;\;\;\;\;\;\;\;\;\;\;(where,y=y\prime ,0\leq z<z\prime +h)\\ & \frac{\ln(\frac{1}{2\tau_{0}}\rho g(z\prime -z))-\alpha ((\frac{l}{v_{rh}}+\frac{h}{v_{rv}})(\left\lfloor \frac{y+l\left\lfloor z/h\right\rfloor }{l}\right\rfloor -\left\lfloor \frac{(\left\lfloor (y+l\left\lfloor z/h\right\rfloor +l_{s})/l_{s}\right\rfloor -1)l_{s}}{l}\right\rfloor )+\frac{y+l\left\lfloor z/h\right\rfloor -(\left\lfloor (y+l\left\lfloor z/h\right\rfloor +l_{s})/l_{s}\right\rfloor -1)l_{s}}{v_{ex}})}{(\frac{l}{v_{rh}}+\frac{h}{v_{rv}})(\left\lfloor \frac{y\prime +l\left\lfloor z\prime /h\right\rfloor }{l}\right\rfloor -\left\lfloor \frac{y+l\left\lfloor z/h\right\rfloor }{l}\right\rfloor )+\frac{y\prime +l\left\lfloor z\prime /h\right\rfloor -(y+l\left\lfloor z/h\right\rfloor )}{v_{ex}}}\;\;\;\;\;\;\;\;\;\;\;\;\;\;\;\;\;\;\;\;\;\;\;\;\;\;\;\\ & \;\;\;\;\;\;\;\;\;\;\;\;\;\;\;\;\;\;\;\;\;\;\;\;\;\;\;\;\;\;\;\;\;\;\;\;\;\;\;\;\;\;\;\;\;\;\;\;\;\;\;\;\;\;\;\;\;\;\;\;\;\;\;\;(where,y\prime <y\leq l,0\leq z<z\prime )\\ 0 & \;\;\;\;\;\;\;\;\;\;\;\;\;\;\;\;\;\;\;\;\;\;\;(where,0\leq y\leq y\prime ,z=z\prime +h\;\;or\;\;y\prime <y\leq l,z\prime \leq z\leq z\prime +h) \end{array}$$

(28b) $$\beta (y,z,y\prime ,z\prime )\geq \{\begin{array}{cc} & \frac{\frac{1}{2}\rho g(z\prime +h-z)-\tau_{0}-\alpha ((\frac{l}{v_{rh}}+\frac{h}{v_{rv}})(\left\lfloor \frac{y+l\left\lfloor z/h\right\rfloor }{l}\right\rfloor -\left\lfloor \frac{(\left\lfloor (y+l\left\lfloor z/h\right\rfloor +l_{s})/l_{s}\right\rfloor -1)l_{s}}{l}\right\rfloor )+\frac{y+l\left\lfloor z/h\right\rfloor -(\left\lfloor (y+l\left\lfloor z/h\right\rfloor +l_{s})/l_{s}\right\rfloor -1)l_{s}}{v_{ex}})}{(\frac{l}{v_{rh}}+\frac{h}{v_{rv}})(\left\lfloor \frac{y\prime +l\left\lfloor z\prime /h\right\rfloor }{l}\right\rfloor -\left\lfloor \frac{y+l\left\lfloor z/h\right\rfloor }{l}\right\rfloor )+\frac{y\prime +l\left\lfloor z\prime /h\right\rfloor -(y+l\left\lfloor z/h\right\rfloor )}{v_{ex}}}\\ & \;\;\;\;\;\;\;\;\;\;\;\;\;\;\;\;\;\;\;\;\;\;\;\;\;\;\;\;\;\;\;\;\;\;\;\;\;\;\;\;\;\;\;\;\;\;\;\;\;\;\;\;\;\;\;\;\;\;(where,0\leq y<y\prime ,0\leq z<z\prime +h)\\ & \frac{\frac{1}{2}\rho g(z\prime +h-z)+\tau_{ex}-\tau_{0}-\alpha ((\frac{l}{v_{rh}}+\frac{h}{v_{rv}})(\left\lfloor \frac{y+l\left\lfloor z/h\right\rfloor }{l}\right\rfloor -\left\lfloor \frac{(\left\lfloor (y+l\left\lfloor z/h\right\rfloor +l_{s})/l_{s}\right\rfloor -1)l_{s}}{l}\right\rfloor )+\frac{y+l\left\lfloor z/h\right\rfloor -(\left\lfloor (y+l\left\lfloor z/h\right\rfloor +l_{s})/l_{s}\right\rfloor -1)l_{s}}{v_{ex}})}{(\frac{l}{v_{rh}}+\frac{h}{v_{rv}})(\left\lfloor \frac{y\prime +l\left\lfloor z\prime /h\right\rfloor }{l}\right\rfloor -\left\lfloor \frac{y+l\left\lfloor z/h\right\rfloor }{l}\right\rfloor )+\frac{y\prime +l\left\lfloor z\prime /h\right\rfloor -(y+l\left\lfloor z/h\right\rfloor )}{v_{ex}}}\\ & \;\;\;\;\;\;\;\;\;\;\;\;\;\;\;\;\;\;\;\;\;\;\;\;\;\;\;\;\;\;\;\;\;\;\;\;\;\;\;\;\;\;\;\;\;\;\;\;\;\;\;\;\;\;\;\;\;\;\;\;\;\;\;\;(where,y=y\prime ,0\leq z<z\prime +h)\\ & \frac{\frac{1}{2}\rho g(z\prime -z)-\tau_{0}-\alpha ((\frac{l}{v_{rh}}+\frac{h}{v_{rv}})(\left\lfloor \frac{y+l\left\lfloor z/h\right\rfloor }{l}\right\rfloor -\left\lfloor \frac{(\left\lfloor (y+l\left\lfloor z/h\right\rfloor +l_{s})/l_{s}\right\rfloor -1)l_{s}}{l}\right\rfloor )+\frac{y+l\left\lfloor z/h\right\rfloor -(\left\lfloor (y+l\left\lfloor z/h\right\rfloor +l_{s})/l_{s}\right\rfloor -1)l_{s}}{v_{ex}})}{(\frac{l}{v_{rh}}+\frac{h}{v_{rv}})(\left\lfloor \frac{y\prime +l\left\lfloor z\prime /h\right\rfloor }{l}\right\rfloor -\left\lfloor \frac{y+l\left\lfloor z/h\right\rfloor }{l}\right\rfloor )+\frac{y\prime +l\left\lfloor z\prime /h\right\rfloor -(y+l\left\lfloor z/h\right\rfloor )}{v_{ex}}}\\ & \;\;\;\;\;\;\;\;\;\;\;\;\;\;\;\;\;\;\;\;\;\;\;\;\;\;\;\;\;\;\;\;\;\;\;\;\;\;\;\;\;\;\;\;\;\;\;\;\;\;\;\;\;\;\;\;\;\;\;\;\;\;\;\;(where,y\prime <y\leq l,0\leq z<z\prime )\\ 0 & \;\;\;\;\;\;\;\;\;\;\;\;\;\;\;\;\;\;\;\;\;\;\;(where,0\leq y\leq y\prime ,z=z\prime +h\;\;or\;\;y\prime <y\leq l,z\prime \leq z\leq z\prime +h) \end{array}$$

**Figure 9:**
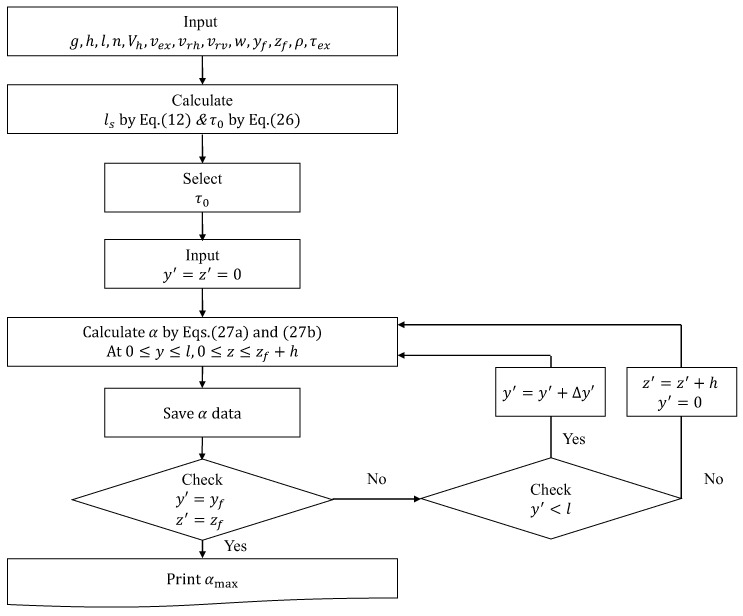
Computational procedures to calculate $\tau_{0}$, $\alpha_{\max}$.

**Figure 10:**
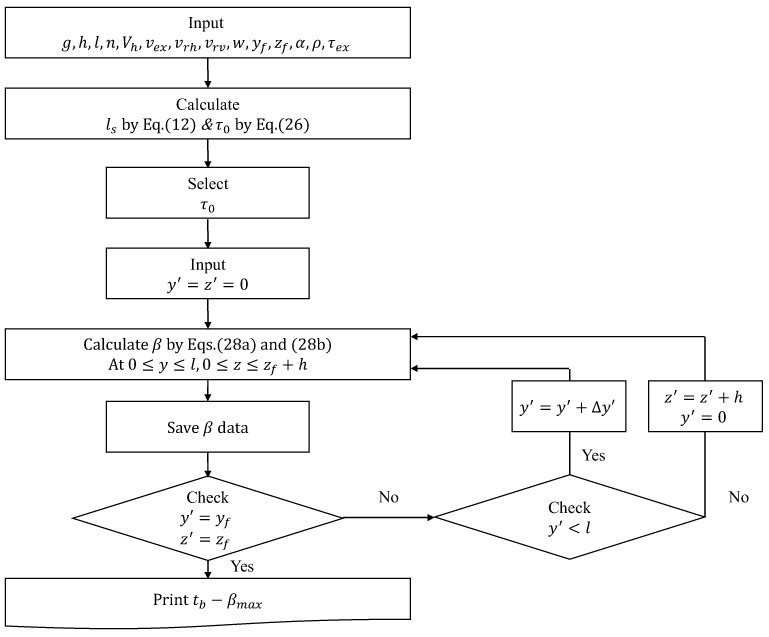
Computational procedures to calculate $\tau_{0}$, $\beta_{\max}$.

Based on Equations (27a) and (27b), the α(y,z,y′,z′) required to prevent collapse and deformation can be calculated by changing (y′,z′) from the start point of extrusion to the coordinates ($y_{f},z_{f}$) of the end point with respect to all the (y,z) within the area in which fresh concrete is layered. In addition, the α(y,z,y′,z′) should have a larger than maximum value ($\alpha_{\max}$) among all the α values calculated in the printing process based on Equation (27a,b), since the mixture proportions of fresh concrete used in printing are all the same. If the accelerator is injected into fresh concrete, the α should be assumed to be as shown in Equation (28a,b) in order to calculate the β(y,z,y′,z′). Even if fresh concrete with the same mixture proportion is used in the build-up process, the β(y,z,y′,z′) may vary depending on the accelerator injection time ($t_{b}$). Therefore, the maximum value ($\beta_{\max}(y,z)$) among the β(y,z,y′,z′) values calculated from the arbitrary (y,z) is calculated based on the β(y,z,y′,z′) values calculated using Equation (28a,b), and represented on the $t_{b}-\beta_{\max}$ plane and the mixture design should be done so that β of fresh concrete can always have $\beta_{\max}$ or more. Figure 9 and Figure 10 show algorithms for calculating $\tau_{0}$ and $\alpha_{\max}$ when the accelerator is not injected into the fresh concrete and $\tau_{0}$ and $\beta_{\max}$ when the accelerator is injected, respectively.

### 3.6. Determination of Analysis Parameters

Table 1 shows the summary of variables for two example cases that are selected for the validation purpose of the proposed model. The size of the 3D printed structure and the width and height of each layer were selected in the consideration of the time required for the CFD analysis. The main variable is the hardening coefficient (β) that changes with the accelerator input and the other properties in Cases 1 and 2 were assumed to be the same. From Equation (26), the $\tau_{0}$ value, which is the minimum yield stress to prevent collapse of a layer while the fresh concrete of upper layer is being discharged, is calculated as 122.5 Pa. According to Petit [19], the hardening coefficient (α) of self-compacting concrete and mortar depends on the proportions of the components of the mixture or temperature of the fresh concrete, ranging from 0.00013 to 0.00025/s. In this example, the α value was assumed to be 0.0003/s based on the fact that the hardening rate of printable concrete is higher than that of the self-compacting concrete. In addition, $\tau_{0}$ was set to 500 Pa that is higher than 122.5 Pa, which was determined to induce the occurrence of deformation immediately after a predetermined number of layers are built up.

**Figure 11:**
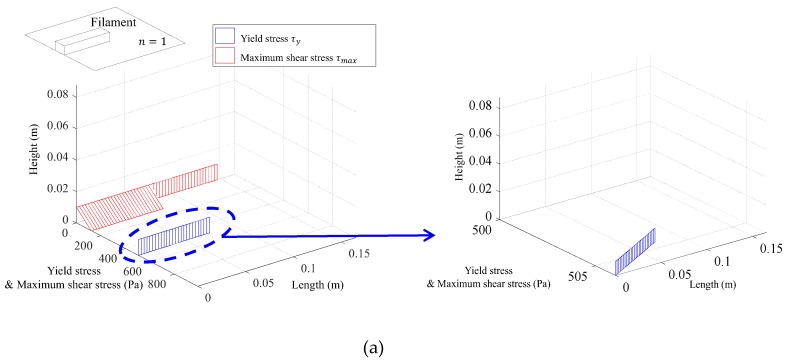

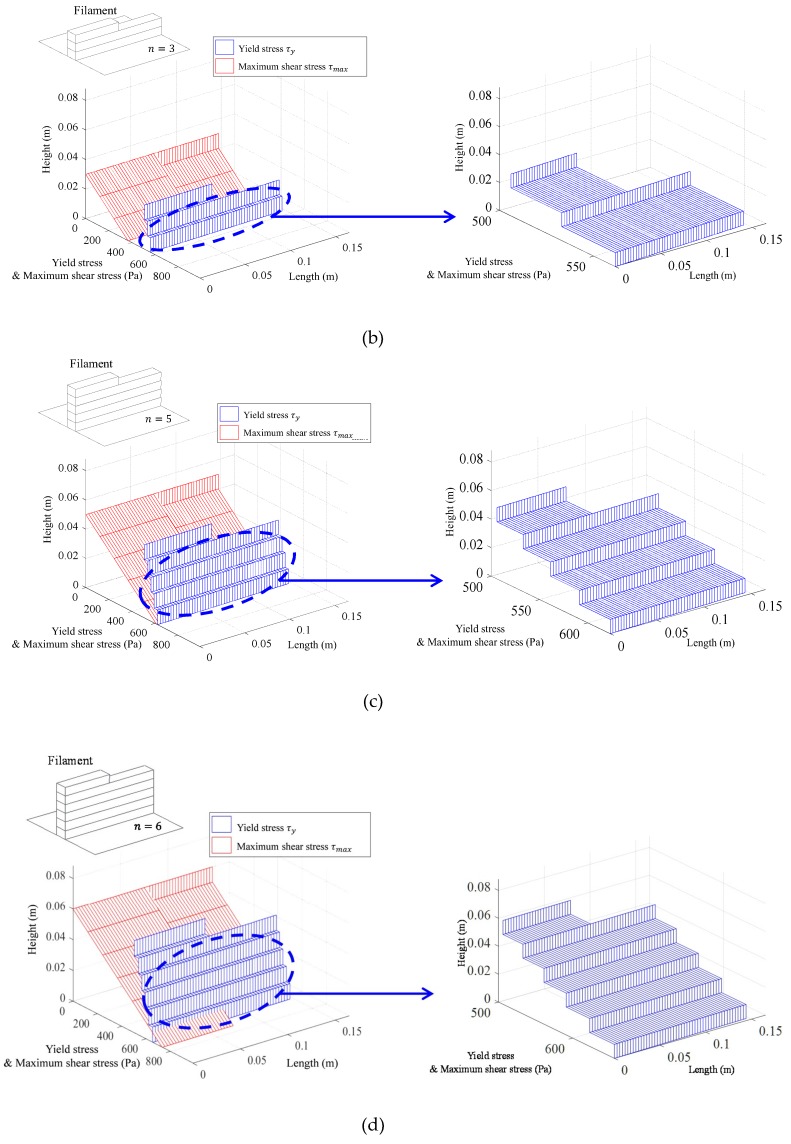

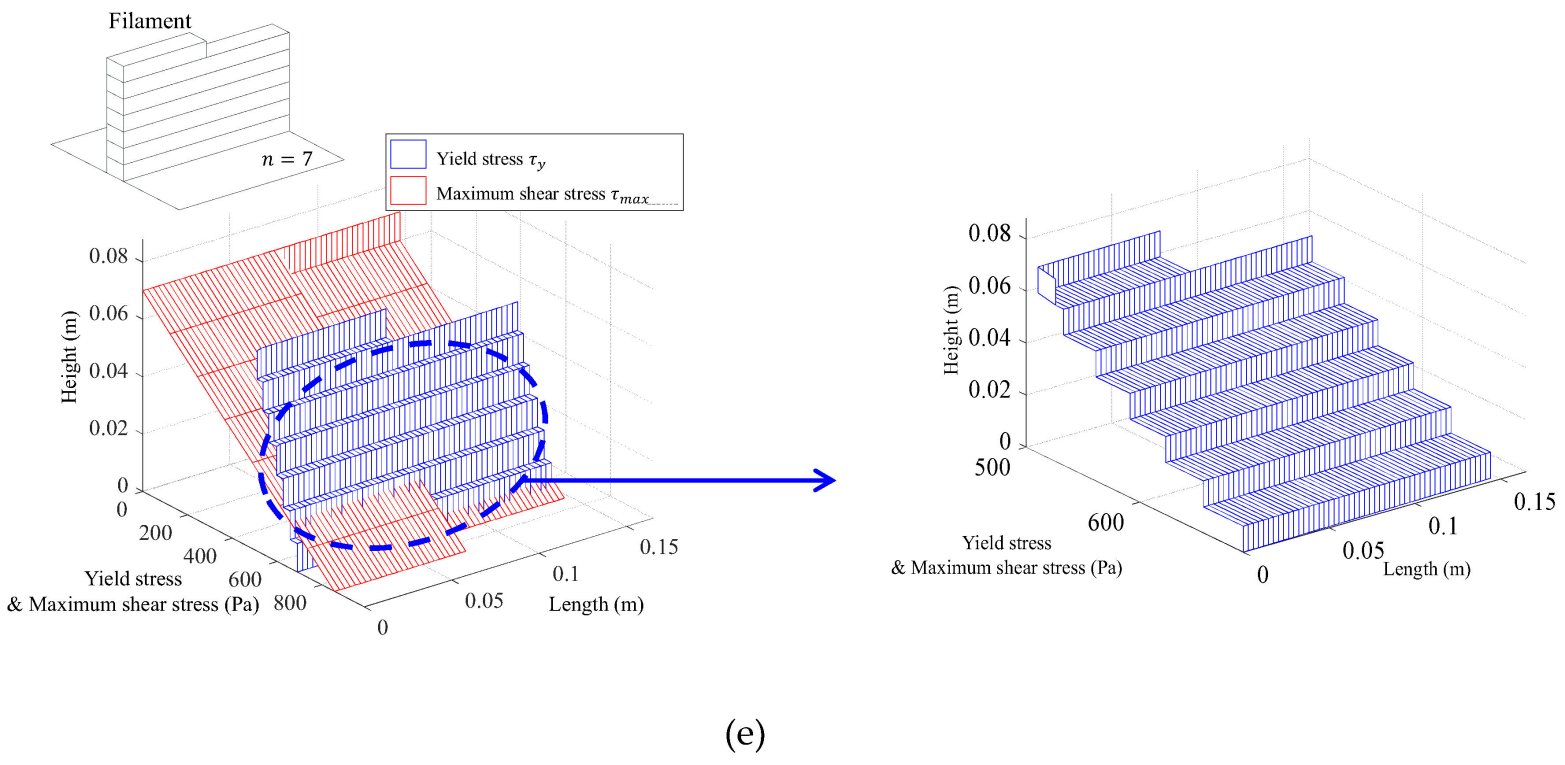
Comparison of yield stress and maximum shear stress. (**a**) Maximum shear stress & yield stress at n=1; (**b**) Maximum shear stress & yield stress at n=3; (**c**) Maximum shear stress & yield stress at n=5; (**d**) Maximum shear stress & yield stress at n=6; (**e**) Maximum shear stress & yield stress at n=7.

Figure 11 shows the comparison between the maximum shear stress distribution and the yield stress distribution analysed using variables corresponding to Case 1 of Table 1, during which the coordinates of the printing end point are ($y_{f},z_{f}$). In Figure 11, the red line indicates the maximum shear stress (i.e., shear demand) due to layering of the extruded concrete and the blue line indicates the yield stress (i.e., shear capacity). The demand curve and the capacity curve do not meet each other until the time at which the fifth layer is built up so that deformation does not occur in the layered concrete. When the sixth layer is built up, the deformation of the concrete in the bottom surface occurs when the maximum shear stress exceeds the yield stress.

**Figure 12:**
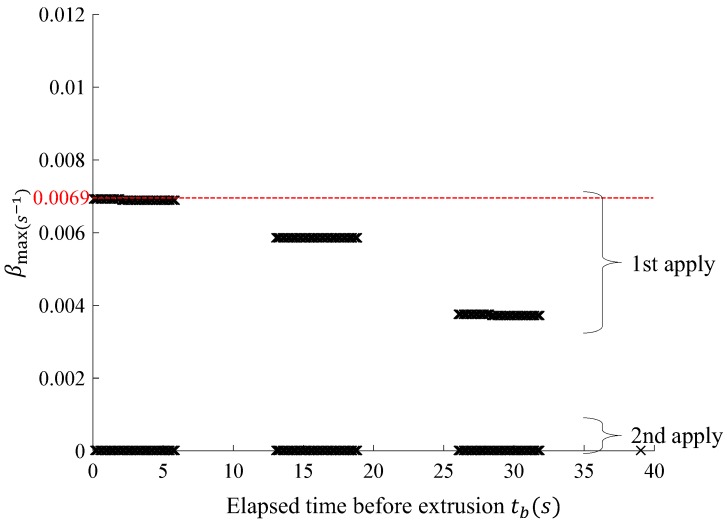
$t_{b}-\beta_{\max}$ curve.

Figure 12 shows the calculation result of $\beta_{\max}$, which does not cause the collapse of fresh concrete during the build-up process under the same printing conditions as found in Case 1, according to the elapsed time before extrusion ($t_{b}$) with the use of the algorithm shown in Figure 10. The analytical results obtained through the proposed model show that the collapse of the fresh concrete does not occur when the $\beta_{\max}$ is greater than 0.0069/s and the value β was set to 0.0069/s in Case 2. In the following section, CFD analysis was performed for Cases 1 and 2 and the reasonability was verified through a comparison between the proposed model and analysis results.

### 3.7. Validation Using Computational Fluid Dynamic Analysis

Computational fluid dynamics (CFD) is one of the analytical techniques used to predict the behaviour of fluids. It has been widely used to simulate the behaviour of fresh concrete analysis [29]. In this study, ANSYS CFX, a commercial software program for numerical analysis, was used to perform a CFD analysis in order to verify the reasonability of the proposed algorithm. The analysis parameters used in the verification are Cases 1 and 2, shown in Table 1.

**Figure 13:**
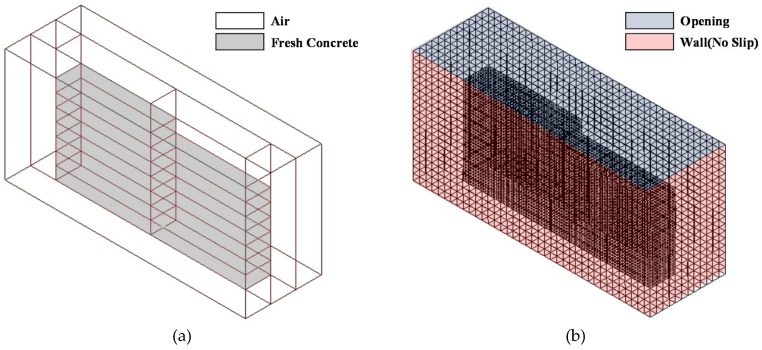
Modelling for CFD analysis. (**a**) Domain of model; (**b**) Mesh and boundary condition.

Figure 13 shows the modelling for the CFD analysis. As shown in Figure 13a, the layered printed concrete consists of the air domain and the fresh concrete domain, while a rectangular mesh composed of 41,924 nodes and 30,698 elements was used as shown in Figure 13b. As for the boundary conditions, an opening was applied to the upper part of the model and a wall (i.e., no slip condition) to the other sides. The modelling also ensured that the force of gravity acts on the whole domain and the Bingham model was used in the CFD analysis. The yield stress value obtained when the analysis was performed under the conditions of Cases 1 and 2, shown in Table 1, was applied with respect to the yield stress ($\tau_{0}$).

**Figure 14:**
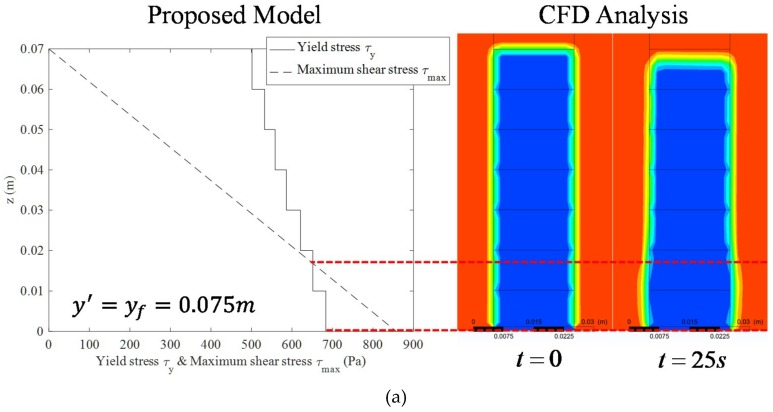

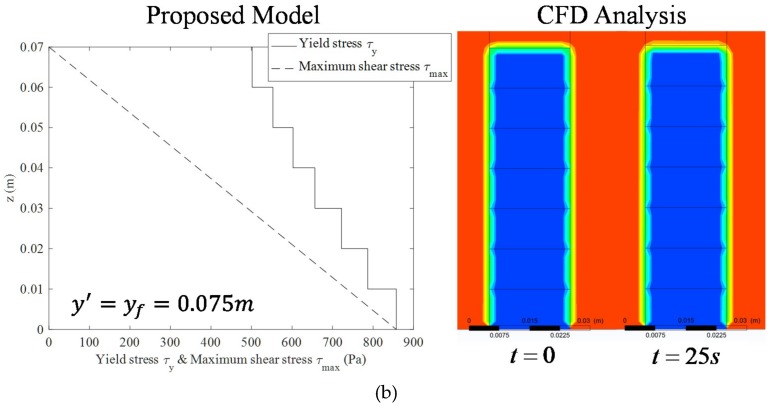
Validation of proposed model. (**a**) Comparison between proposed model and CFD analysis (case 1); (**b**) Comparison between proposed model and CFD analysis (case 2).

Figure 14 shows the comparison between the proposed model and the CFD analysis results for Cases 1 and 2 in Table 1. The left graphs of Figure 14a,b show the yield stress distribution and the maximum shear stress distribution in the case of $y\prime =y_{f}=0.075\;m$ in the proposed model. It should be noted that in Cases 1 and 2, the other properties are the same except for the hardening coefficient (*β*), due to the injection of accelerator. The *β* of Case 2 is the hardening coefficient, which is calculated through the algorithm proposed in this study required to prevent the collapse of fresh concrete. In Case 1, the proposed model well predicted the CFD simulation results, in which the fresh concrete collapsed at a height of approximately 0.018 m. On the other hand, in Case 2, the fresh concrete did not collapse in both results by the proposed model and the CFD analysis.

## 4. Conclusion

This study aimed to develop an algorithm to quickly derive the rheological properties needed to prevent the collapse of fresh concrete during the printing process. In the proposed model, the fresh concrete was regarded as Herschel-Bulkley fluid and it was thus assumed that no deformation occurs before the maximum shear stress of layered concrete exceeds its yield stress. The maximum shear stress in the layered fresh concrete was calculated from the force equilibrium condition and the yield stress of the fresh concrete at any location during the printing process was estimated considering the elapsed time that depends on the printing path. Based on this research, the following conclusions were drawn:

1. The proposed model could reflect the increase in the yield stress of the fresh concrete during the 3D concrete printing process by substituting the elapsed time into the yield stress-elapsed time equation.
2. Based on the CFD analysis results on the cases examined in this study, it was confirmed that the proposed model can provide very accurate estimations on the occurrence and location of collapse.
3. The proposed model would be very helpful to obtain the rheological properties of fresh concrete required for 3D printing, such as hardening coefficient and initial yield stress, without any complex numerical simulations.
4. Further researches are still required to check the stability problems of the layered concrete for vertically complicated structures.

## Figures and Tables

**Table 1:** Summary of printing properties for analysis.

Information	Case 1	Case 2
Injection of accelerator	Applied	
$\mathrm{Shape}\;\mathrm{of}\;t_{e}-\tau_{y}$ curve	Exponential	
g	9.8 m/s^2^	
h	0.01 m	
$l,l_{i}$	0.15 m	
n	7	
$V_{h}$	0.00003	
$v_{ex},v_{rh}$	0.025 m/s	
$v_{rv}$	0.01 m/s	
w	0.02 m	
$y_{f}$	0.075 m	
$z_{f}$	0.06 m	
ρ	2500 kg/m^3^	
$\tau_{ex}$	0	
$\tau_{0}$	500 Pa	
α	0.0003/s	
β	0.004/s	0.0069/s
